# Prevalence and factors that influence smokeless tobacco use among adults in pastoralist communities of Borena Zone, Ethiopia: mixed method study

**DOI:** 10.1186/s12971-016-0106-7

**Published:** 2017-01-05

**Authors:** Edao Sinba Etu, Desta Hiko Gemeda, Mamusha Aman Hussen

**Affiliations:** 1 Negele Borena Health Science College, Oromia Regional State, Negele Borena, Ethiopia; 2 College of Health Sciences, Department of Epidemiology, Jimma University, Jimma, Ethiopia; 3 College of Health Sciences, Department of Health Education and Behavioral Sciences, Jimma University, Jimma, Ethiopia

**Keywords:** Tobacco, Smokeless, Adult, Pastoralist, Ethiopia

## Abstract

**Background:**

Deaths due to tobacco consumption are on the rise, from 5.4 million in 2005 to 6.4 million in 2015 and 8.3 million in 2030 of which more than 80% will be in developing countries. Smokeless tobacco use is a significant health risk and cause of disease. Over 300 million people use smokeless tobacco worldwide. More than 250 million adult smokeless tobacco users are in low- and middle-income countries, the total burden of smokeless tobacco use is likely to be substantial. In Ethiopia, nationally representative data on the smokeless tobacco use is not available. Most studies conducted in the country focused on cigarette smoking.

**Method:**

A community based cross-sectional study using quantitative and qualitative approaches was conducted from September 14–29, 2015. The study was conducted among adults in pastoralist communities in Borena zone, Ethiopia. A total of 634 households were selected randomly for interview. An interviewer-administered questionnaire and in-depth interview guide was used to assess adults’ practice, attitude, knowledge, and perception on Smokeless Tobacco use. Logistic regression was used to assess association between dependent and independent variables.

**Result:**

Out of 634 participants, 287 (45.3%) of them were current users of smokeless tobacco. Being Muslim (AOR = .21, 95% CI: .13, .33), being Christian (AOR = .38, 95% CI: .22, .67), and having good health risk perception toward smokeless tobacco use (AOR = .49, 95% CI: .34, .70) were protective factors for smokeless tobacco use, whereas favorable attitude (AOR = 2.12, 95% CI: 1.48, 3.04) and high social pressure towards smokeless tobacco use (AOR = 1.73, 95% CI: 1.21, 2.47) were factors independently associated with smokeless tobacco use.

**Conclusion:**

This study concludes that smokeless tobacco use is very common in the selected districts of the Borena zone. The practice is strong linked to the lifestyle of the community.

## Background

Tobacco use is one of the leading preventable causes of early death, disease, and disability around the world [1]. Tobacco is the second major cause of death in the world. Every 6.5 s one tobacco user dies from a tobacco-related disease some-where in the world [2]. Annually, an estimated 4.9 million deaths occurring worldwide attributed to tobacco consumption [3]. Deaths due to tobacco consumption are on the rise, from 5.4 million in 2005; and projected to rise to 6.4 million in 2015 and 8.3 million in 2030 of which more than 80% will be in developing countries [4].

In Sub-Saharan Africa, tobacco use caused just 100,000 deaths in 1990 and is projected to lead to deaths of more than 700,000 people in 2015 [5]. As a result, low and middle-income countries are expected to experience a doubling of deaths attributable to tobacco from 3.4 million to 6.8 million [6]. Tobacco use is responsible for 1.4 million cancer deaths per year. Lung, oral, and nasopharyngeal cancers are some of the major cancers caused by tobacco consumption [7, 8].

Discussions of disease and health in Africa and other low- and middle-income countries (LMICs) often focus on infectious diseases. However, according to the WHO, chronic diseases exert a huge toll, with in low and middle income countries (LMICs) contributing 80% of global non-communicable disease deaths, including those resulting from tobacco use and second-hand smoke exposure [4].

In addition to health effect, the use of tobacco adds a burden to the national economy by increasing costs in health expenditure and other indirect costs related to illness due to tobacco borne diseases [9]. Thus, aggravate poverty and hold back economic development by leaving families with less money to spend on such basic items as food and education [10].

World Health Organization (WHO) estimated an Ethiopian national tobacco use prevalence at 7.6% [11], while recent study reported adolescents prevalence of 17.2%, (13.3% males and 3.8% females) in 2014 [12]. Most of these studies indicate cigarette smoking have become common practices among high school students [13, 14] and college/university students in Ethiopia [15, 16].

Smokeless tobacco use is a significant health risk and cause of disease [17]. Over 300 million people use smokeless tobacco worldwide. More than 250 million adult ST users are in low- and middle-income countries, the total burden of smokeless tobacco use is likely to be substantial [18, 19]. Unlike cigarettes and other forms of tobacco, smokeless tobacco is not burned. Instead, nicotine is absorbed into the body through direct contact of the tobacco with mucous membranes in the mouth or nose. In addition to nicotine, smokeless tobacco contains over 3000 chemicals, [20], including 28 known carcinogens (cancer-causing compounds [21].

The amount of nicotine absorbed when using smokeless tobacco is two to three times the amount that someone gets from a cigarette. A person who consumes eight to ten dips or chews per day receives the same amount of nicotine as someone who smokes 30 to 40 cigarettes er day [20]. The average cigarette contains 8.4 mg of nicotine while an average “dip” of moist snuff has 14.5 mg of nicotine, meaning someone using chewing tobacco can be exposed to as much as 133 mg of nicotine in a day [21]. Moreover, smokeless tobacco use in some regions appears concurrently with cigarette smoking, thus contributing to the total health burden of tobacco use [19, 22].

Yet international tobacco control efforts have largely focused on cigarettes, devoting only limited attention to other types of products, including smokeless tobacco. Reduction in the prevalence of tobacco smoking in many developed countries, forces tobacco companies to look for opportunities to develop new growth in tobacco use in developing counties and began to market new products in smokeless form [23, 24] consequently, the use of smokeless tobacco is growing in popularity [25].

Data from the Global Youth Tobacco Survey show that students aged 13–15 surveyed in 132 countries were more likely to report using non-cigarette tobacco products including smokeless tobacco products (11.2%) than to report smoking cigarettes (8.9%) [26, 27]. A secondary data study that was undertaken in Congolese school-going adolescents, found that the prevalence of smokeless tobacco use was 18.0%, with no sex or age differences in the prevalence [28]. However, in Ethiopia, nationally representative data on prevalence and the effects of smokeless tobacco use are not available [29]. Most of studies conducted in the country focused on cigarette smoking among urban populations or students [30–33].

Our study aimed to fill a literature gap on the prevalence and health effects of smokeless tobacco use in a region of Ethiopia. We would like to illustrate that even though, trends in prevalence of smokeless tobacco use and its health effect have not been well studied, there are several adverse health effects attributable to smokeless tobacco use [3, 34].

## Methods

### Study area

The study was carried out among pastoralists in the Borena zone of the Oromia Regional State, Ethiopia, from September 14–29, 2015. Borena zone is one of the largest pastoralists among pastoralist areas in Oromia regional state. The zone has an estimated total population of 878,161 and 162,746 households according to Central Statistical Agency [11].

### Participants and sampling strategies

Multistage sampling technique was used to recruit study participants. Borena zone was selected on purposive because its remoteness and large size compared to other pastoralist zones. Simple random sampling was used to select three districts out of ten pastoralist districts namely; Yabello, Arero and Moyale districts were selected. Then, simple random sampling was used to select 30% of kebeles (smallest administrative unit) in each districts (total 20 kebeles from three districts were included in the study) that was seven kebeles from Yabello, seven kebeles from Arero and six Moyale districts. Sampling frame of Household was prepared from family folders in selected kebeles. Then, the sample size was allocated proportional to the size of households in each selected kebeles. Finally, the simple random sampling technique was used to select individuals for the interview (Table 1).

**Table 1:** Selected districts with their total population, estimated households and proportional allocation, Borena zone, Oromia Regional State, Southern Ethiopia, 2015

Sr.	Districts	Total Kebeles	Population	HHs (/4.8 conversion factor)	No of Selected Kebeles	No of HHs in selected kebeles	PPA of HHs from selected kebeles
1	Yabello	23	104,743	21822	7	9788	140
2	Arero	21	41,583	8664	7	4125	59
3	Moyale	20	159,499	33228	6	12952	186
Total		64	305,825	63,714	20	26,865	634

*PPA* Proportional Allocation

During the data collection process, closed houses were revisited for three times. The persons who were not available after they were selected for the study were considered as non-respondents. For more than one person fulfilling the inclusion criteria in the same household, one person was selected using the lottery method.

The sample size was determined by using single population proportion formula considering the following parameters; since there is no similar study on SLT in Ethiopia, sample size was calculated by assuming 50% prevalence of SLT use, 95% confidence level, and 5% margin of error. Thus, final sample size by considering a 10% non-response rate and design effect of 1.5, was 634 households.

The participants for the in-depth interview were selected using purposive sampling technique on the basis of a prior specification of desired characteristics like SLT use, experience level. Adult above 18 years and reside in that community for 6 months and above was included in the interviews.

### Measurement and instrument

The structured questionnaire comprised of five parts: socio-demographic characteristics, smokeless tobacco use, knowledge about SLT health effect, attitude towards smokeless tobacco use, health risk perception, and social norm. The socio-demographic section collected information on the age, marital status, religion, ethnicity, income, education status, and occupation of the respondent. The smokeless tobacco use was measured using an items adapted from Global adult tobacco survey (GATS) [35]. The GATS includes core questions (smokeless tobacco use prevalence, pattern of consumption, and exposure to anti-smokeless message on media).

The knowledge of smokeless tobacco health effect was assessed by multiple choice questions. A correct answer was given one mark, while a wrong answer was given zero. Knowledge scores ranged from 0 to 13 and mean score was used cut off level to classify as insufficient knowledge and sufficient knowledge. Attitude was measured by items in which the responses were rated on five point likert scale ranging from (1) strongly disagree to (5) strongly agree. The score of all items were summed and higher score reflect positive attitude towards SLT use. SLT use health risk perception was assessed by items which measure respondents perception on chance of occurrence of a health risk and perceived severity of health consequences. The items were rated on five point likert scale. The score of all items were summed and higher score reflect higher risk perception. Social norms for SLT use was measure by items that assess what respondents believe happening in their neighborhood or community or what respondent perceived other people in their neighborhood do and extent to which respondents believe that they expected to use SLT or get approval from others. Items, asking respondents to indicate their agreement with a number of statements on a 5-point likert scale with responses ranging from ‘strongly disagree’ to ‘strongly agree’. The score of all items will be summed and higher score reflects higher social towards SLT use.

### Data collection procedures

Prior to field implementation of data collection, twelve research assistants and two supervisors were trained on their role, responsibilities, purpose of the study, contents of questionnaires, data collection techniques, and data recording techniques and questionnaire was pretested. Data were collected using structured questionnaire through face to face interview in local language (Afan Oromo). To ensure quality of data, the following measures were undertaken: The questionnaire, which was prepared in English, was translated to Afan Oromo and back translated to English by a translator who was blinded to the original questionnaire prepared in English to check consistency.

### Statistical analysis

Collected data were entered into Epi-Data version 3.1 and exported to SPSS version 20.0 for analysis. Descriptive statistics were performed and presented by text, tables and graphs. Chi-squared test was used to determine adequacy of the cells and test the association between independent variables and the outcome. Binary logistic regression was used to examine the relationship between the proposed predictors and SLT use. Variables with *p*-value < 0.25 were selected for multivariable logistic regression analysis to identify factors independently associated with the outcome. Odds ratio was used as measure of strength of association (with the accompanying *p*-values and confidence intervals). A *p*-value less than 0.05 was used as statistical significance.

For qualitative part, trained and experienced research assistants conducted audio-taped based interview and notes were taken to ensure completeness of data. The verbatim was transcribed first in to language in which the interview was conducted [Afan Oromo] then translated to English by research assistants. The principal investigator validated the transcript and verbatim.

The interview was audio-taped based on willingness of respondents and notes were taken to ensure completeness of data.

After validating the transcription, the typed narratives were then translated into English. The principal investigator and supervisors conducted analysis of data using thematic analyses aiming to identify a set of main themes that captured the diverse views and feelings expressed by respondents. The transcripts were reviewed many times, and codes were developed to describe groups of words or categories with similar meanings. The categories were identified and used to generate themes emerging from the data. Direct quotations of key informants were presented in key findings to triangulate with quantitative findings.

### Ethical consideration

The ethical clearance was obtained from the research ethics committee of Jimma University. Permission was obtained from Oromia Regional State and from administrative bodies of each zone and districts including kebeles. Verbal consent was obtained from each respondent after explaining the purpose, benefit, the confidentiality and voluntary participation features of the study. Moreover, the study questionnaire was anonymous and interview was conducted in a private setting to maintain privacy of the respondents for sensitive questions.

## Results

### Socio-demographic characteristics

In this study, 634 (100%) adults participated in the study. Of the total respondents, 414 (65.3%) were male, 542 (85.5%) were married, 399 (62.9%) were *Wakefata,* 630 (99.4%) were from Oromo ethnic group, 502 (79.2%) were cannot read and write, 490 (77.3%) were pastoralist, 291 (45.9%) had an income range between 151 and 650 (which is approximately 7–31 USD) per month and 140 (22.1%) were in the age group >50 years with a mean age of 42.24 (Table 2).

**Table 2:** Socio-demographic characteristics of the study participants, Borena zone, Oromia Regional State, Southern Ethiopia, 2015

Characteristics of respondents		Number	Percent
Age category	Mean ± SD age (years)	42.24 ± 16.2	
	<25	81	12.8
	25–30	99	15.6
	31–35	75	11.8
	36–40	112	17.7
	41–45	62	9.8
	46–50	65	10.3
	>50	140	22.1
Sex	Male	414	65.3
	Female	220	34.7
Religion	Wakefata	399	62.9
	Muslim	162	25.6
	Christian	73	11.5
Ethnicity	Oromo	630	99.4
	Others^a^	4	.6
Educational level	Cannot read and write	502	79.2
	Read and write but no formal education	108	17.0
	At least primary	24	3.8
Current marital status	Single	41	6.5
	Married	542	85.5
	Divorced	26	4.1
	Widowed	25	3.9
Occupation	Pastoralist	490	77.3
	Others^b^	144	22.7
Income category	0–150	269	42.4
	151–650	291	45.9
	651–1400	69	10.9
	1401–2350	5	.8

^a^Amhara, konso

^b^Agro pastoralist, merchants

### Prevalence of smokeless tobacco use

In this study, of all of respondents 287 (45.3%) of participants were current smokeless tobacco users.

*One of the in-depth interview participants explained as below;*

*“Many people use smokeless tobacco in the villages; both men and women chew tobacco.”*

Half of participants 144 (50.2%) who were current smokeless tobacco users reported that one of their family members were smokeless tobacco users. The majority of participants 231 (80.5%) who were current smokeless tobacco users reported that their close friends were smokeless tobacco users.

More than one-fourth of adults 80 (27.9%), who were current smokeless tobacco users were concurrent users of both cigarette and smokeless tobacco. Of all current smokeless tobacco users, 235 (81.9%) of participants were reported that they did not make any quit attempt of smokeless tobacco use in the past 12 months. Only few adults 24 (7%) who were current smokeless tobacco non-users were former smokeless tobacco users. From all current smokeless tobacco users, 195 (67.9%) of participants were reported that they were not advised to quit smokeless tobacco use by health care providers in the past 12 months. For 210 (33.1%) daily smokeless tobacco users, the mean frequency of chewing per day was 8.6 (SD = 3.5); and for 77 (12.1%) occasional smokeless tobacco users, the mean of chewing per week was 5.8 (SD = 8.2) (Table 3).

**Table 3:** Detailed smokeless tobacco use status by Gender, Borena zone, Oromia Regional State, Southern Ethiopia, 2015

Current smokeless tobacco status			Overall		Male	Female
			No	(%)	*N* (%)	*N* (%)
Current smokeless tobacco user	Daily user		210	33.1	131 (20.7)	79 (12.4)
	Occasional user		77	12.1	46 (7.2)	31 (4.9)
Occasional user	Occasional user, formerly daily		25	3.9	12 (1.9)	13 (2.0)
	Occasional user, never daily		52	8.2	34 (5.4)	18 (2.8)
Current non-user of smokeless tobacco	Former daily user		12	1.9	6 (0.9)	6 (0.9)
	Former occasional user		12	1.9	11 (1.7)	1 (0.2)
	Never smokeless user		323	50.9	220 (34.7)	103 (16.2)
For currently daily users: Age at SLT use debut?	<25 years		146	69.5	93 (44.3)	53 (25.2)
	25–30 year		17	8.1	17 (8.1)	0 (0.0)
	>30 year		11	5.2	4 (1.9)	7 (3.3)
	I don’t remember		36	17.1	17 (8.1)	19 (9.0)
For currently daily users: How soon after you wake up do you usually use smokeless tobacco for the first time?	Within 5 min		28	13.3	22 (10.5)	6 (2.9)
	6 to 30 min		103	49.0	70 (33.3)	33 (15.7)
	31 to 60 min		35	16.7	16 (7.6)	19 (9.0)
	More than 60 min		44	21.0	23 (11.0)	21 (10.0)
For currently daily users: On average, how many times a day do you use the following products?	Chewing tobacco	2	4	1.9	2 (1.0)	2 (1.0)
		3	11	5.2	5 (2.4)	6 (2.9)
		4	15	7.1	9 (4.3)	6 (2.9)
		5	13	6.2	7 (3.3)	6 (2.9)
		>5	167	79.5	108 (51.4)	59 (28.1)
For occasional user: How many times a week do you usually use the following products?	Chewing tobacco	2	8	10.4	6 (7.8)	2 (2.6)
		3	25	32.5	18 (23.4)	7 (9.1)
		4	27	35.1	13 (16.9)	14 (18.2)
		5	5	6.5	2 (2.6)	3 (3.9)
		>5	12	15.6	7 (9.1)	5 (6.5)
For currently SLT users: family members using SLT?	Yes		144	50.2	88 (30.7)	56 (19.5)
	No		143	49.8	89 (31.0)	54 (18.8)
For currently SLT users: close friends smoke or chew tobacco?	Yes		231	80.5	148 (51.6)	83 (28.9)
	No		56	19.5	29 (10.1)	27 (9.4)
For currently SLT users: In addition to chewing or snuffing or applying SLT, do you smoke cigarette	Yes		80	27.9	57 (19.9)	23 (8.0)
	No		207	72.1	120 (41.8)	87 (30.3)
For currently SLT users: During the past 12 months, have you tried to stop using smokeless tobacco?	Yes		52	18.1	36 (12.5)	16 (5.6)
	No		235	81.9	141 (49.1)	94 (32.8)
Noticed anti SLT use information in newspapers or in magazines in the last 30 days	Yes		21	3.3	11 (1.7)	10 (1.6)
	No		613	96.7	403 (63.6)	210 (33.1)
Noticed anti SLT use information on television or radio in the last 30 days	Yes		53	8.4	31 (4.9)	22 (3.5)
	No		581	91.6	383 (60.4)	198 (31.2)
Advised by health professionals to stop using smokeless tobacco in past 12 months,	Yes		92	32.1	55 (19.2)	37 (12.9)
	No		195	67.9	122 (42.5)	73 (25.4)

Among male participants, 177 (42.8%) were smokeless tobacco users while half 110 (50%) of female participants were smokeless tobacco users. Among *Wakefata* followers, 232 (58.1%) were smokeless tobacco users. Among divorced participants 15 (57.7%) were smokeless tobacco users. Among age category >50 years participants, 75 (53.6%) were smokeless tobacco users (Table 4).

**Table 4:** Prevalence of smokeless tobacco use by demographic characteristics of respondents, Borena zone, Oromia Regional State, Southern Ethiopia, 2015

Characteristics of respondents		Current smokeless tobacco status	
		Non users(*n* = 347)	Users(*n* = 287)
		No (%)	No (%)
Age category	<25	48 (59.3)	33 (40.7)
	25–30	55 (55.6)	44 (44.4)
	31–35	44 (58.7)	31 (41.3)
	36–40	63 (56.2)	49 (43.8)
	41–45	39 (62.9)	23 (37.1)
	46–50	33 (50.8)	32 (49.2)
	>50	65 (46.4)	75 (53.6)
Sex	Male	237 (57.2)	177 (42.8)
	Female	110 (50)	110 (50)
Religion	Wakefata	167 (41.9)	232 (58.1)
	Muslim	129 (79.6)	33 (20.4)
	Christian	51 (69.9)	22 (30.1)
Ethnicity	Oromo	346 (54.9)	284 (45.1)
	Others^a^	1 (25)	3 (75)
Educational status of mothers	Cannot read and write	267 (53.2)	235 (46.8)
	Read and write but no formal education	66 (61.1)	42 (38.9)
	At least primary	14 (58.3)	10 (41.7)
Current marital status	Single	22 (53.7)	19 (46.3)
	Married	307 (56.6)	235 (43.4)
	Divorced	11 (42.3)	15 (57.7)
	Widowed	7 (28)	18 (72)
Occupation	Pastoralist	269 (54.9)	221 (45.1)
	Others^b^	78 (54.2)	66 (45.8)
Income category	0–150	155 (57.6)	114 (42.4)
	151–650	149 (51.2)	142 (48.8)
	651–1400	40 (58)	29 (42)
	1401–2350	3(60)	2 (40)

^a^Amhara, konso

^b^Agro pastoralist, merchants

Nearly three fourth 246 (70.9%) of adults who were current smokeless tobacco non-users had unfavorable attitude towards smokeless tobacco use while more than half 147 (51.2%) of adults who were current smokeless tobacco users had favorable attitude towards smokeless tobacco use.

50-year-old male SLT user said that*:*

“…*tobacco helped me to get relief from pain; even it helps me to forget issues that worry me. If feel alright only after I use tobacco otherwise I feel discomfort”*

Majority of current smokeless tobacco non-users 212 (61.1%) and users 170 (59.2%) had insufficient knowledge about smokeless tobacco use health effect.

A 65 years old female SLT user stated that:

*“…personally, I do not know any heath problem caused by using smokeless tobacco. It may cause discoloration of teeth; of course, this is not a big problem. Rather it serves as painkiller for teeth and head ache”*

The majority of current smokeless tobacco users 184 (64.1%) had poor health risk perception of smokeless tobacco use.

*In-depth interview respondent indicates that*

*”..I have been using this tobacco for almost 25 years, until now I did not experience any health problem. There is no disease caused by tobacco use rather it helps to cure pain such as teeth ache” [80 years old male SLT user] A 36 year old SLT user and smoker added that*

*“I think using smokeless tobacco is less harmful than smoking cigarettes. I think cigarettes have ‘something’ that makes people addicted to the cigarettes.”*

Majority of current smokeless tobacco non-users 228 (65.7%) had low social pressure as compared to more than half 162 (56.4%) of current smokeless tobacco users who were reported that they had high social pressure towards smokeless tobacco use (Table 5).

**Table 5:** Knowledge, Attitudes, Health risk perception and Social norm characteristics of Respondents by category, Borena zone, Oromia Regional State, Southern Ethiopia, 2015

Characteristics		Current smokeless tobacco status			
		Non users (*n* = 347)		Users (*n* = 287)	
		N0	(%)	N0	(%)
Attitude towards smokeless tobacco use	Unfavorable	246	70.9	140	48.8
	Favorable	101	29.1	147	51.2
Knowledge about SLT health effect	Insufficient knowledge	212	61.1	170	59.2
	Sufficient knowledge	135	38.9	117	40.8
Health risk perception	Poor perception	188	54.2	184	64.1
	Good perception	159	45.8	103	35.9
Social norm	Low social pressure	228	65.7	125	43.6
	High social pressure	119	34.3	162	56.4

*A 48 year old in depth interview participant stated that*

*“My grand-mother showed me how to prepare and chew tobacco every time I stayed with her. She also said that it would help to avoid headache, mouth and teeth diseases.”*

*“I have chewed tobacco for about ten years, I chew for my pleasure, when I have a tobacco after my food it makes my meal fantastic. My sons are not chewers and they blame me and have asked me not to chew. It makes me nervous with them sometimes and I reply to them that we are in a modern society, I can chew, and if chewing is bad why do the developed countries and people in our country produce smokeless tobacco, if they do not want people to chew?” –65-years –old male SLT user*

### Factors associated with smokeless tobacco use (Bivariate analyses)

Female (*p* = .08), >50 years age groups (*p* = .07), Muslim (*p* = <.0001), Christian (*p* = <.0001), adult education: Read and write but no formal education (*p* = .13), single (*p* = .05), married (*p* = .01), attitude: favorable (*p* = <.0001), Health risk perception: Good perception (*p* = .01) and Social norm: High social pressure (*p* = <.0001) variables were statistically significant on bivariate analysis (Table 6).

**Table 6:** Factors associated with smokeless tobacco use, Borena zone, Oromia Regional State, Southern Ethiopia, 2015

Characteristics of respondents		Current smokeless tobacco status		COR (95% CL)	Adjusted OR (95% CL)
		Non users (*n* = 347)	Users (*n* = 287)		
		No (%)	No (%)		
Age category	<25	48 (13.8)	33 (11.5)	1	
	25–30	55 (15.9)	44 (15.3)	1.16 (0.64,2.11)	
	31–35	44 (12.7)	31 (10.8)	1.03 (0.54,1.94)	
	36–40	63 (18.2)	49 (17.0)	1.13 (0.63,2.02)	
	41–45	39 (11.2)	23 (8.0)	.86 (0.44,1.69)	
	46–50	33 (9.5)	32 (10.3)	1.41 (0.73,2.72)	
	>50	65 (18.7)	75 (26.1)	1.68 (0.97,2.92)	
Sex	Male	237 (68.3)	177 (61.7)	1	
	Female	110 (31.7)	110 (38.3)	1.34 (0.96, 1.86)	
Religion	Wakefata	167 (48.1)	232 (80.8)	1	1
	Muslim	129 (37.2)	33 (11.5)	.84 (.12, .28)*	.21 (.13, .33)
	Christian	51 (14.7)	22 (7.7)	.31 (.18, .53)*	.38 (.22, .67)
Ethnicity	Oromo	346 (99.7)	284 (99.0)	1	
	Others*@	1 (0.3)	3 (1.0)	3.66 (.38, 35.31)	
Educational status of mothers	Cannot read and write	267 (76.9)	235 (81.9)	1	
	Read and write but no formal education	66 (19.0)	42 (14.6)	.72 (.47, 1.11)	
	At least primary	14 (4.0)	10 (3.5)	.81 (.35,1.86)	
Current marital status	Single	22 (6.3)	19 (6.6)	.34 (.12, .98)	
	Married	307 (88.5)	235 (81.9)	.30 (.12, 0.72)	
	Divorced	11 (3.2)	15 (5.2)	.53 (.17, 1.71)	
	Widowed	7 (2.0)	18 (6.3)	1	
Occupation	Pastoralist	269 (77.5)	221 (77.0)	1	
	Others**$	78 (22.5)	66 (23.0)	1.03 (.71, 1.50)	
Income category	0–150	155 (44.7)	114 (39.7)	1	
	151–650	149 (42.9)	142 (49.5)	0.91 (0.15, 5.51)	
	651–1400	40 (11.5)	29 (10.1)	0.70 (0.12, 4.23)	
	1401–2350	3 (0.9)	2 (0.7)	0.92 (0.14, 5.86)	
Attitude towards smokeless tobacco use	Unfavorable	246 (70.9)	140 (48.8)	1	1
	Favorable	101 (29.1)	147 (51.2)	2.56 (1.84, 3.55)*	2.12 (1.48, 3.04)
Knowledge about SLT health effect	Insufficient knowledge	212 (61.1)	170 (59.2)	1	
	Sufficient knowledge	135 (38.9)	117 (40.8)	1.08 (.79, 1.49)*	
Health risk perception	Poor perception	188 (54.2)	184 (64.1)	1	1
	Good perception	159 (45.8)	103 (35.9)	.66 (.48, .91)*	.49 (.34, .70)
Social norm	Low social pressure	228 (65.7)	125 (43.6)	1	1
	High social pressure	119 (34.3)	162 (56.4)	2.48 (1.80, 3.43)*	1.73 (1.21, 2.47)

*statistically significant at *p* < 0.05

@Amhara, konso

$Agro pastoralist, merchants

### Factors associated with smokeless tobacco use (Multiple variable analyses)

Religion: being Muslim (AOR = .21, 95% CI: .13, .33) and Christian (AOR = .38, 95% CI: .22, .67), Attitude: favorable attitude towards smokeless tobacco use (AOR = 2.12, 95% CI: 1.48, 3.04), Health risk perception: good health risk perception on smokeless tobacco use (AOR = .49, 95% CI: .34, .70) and social norm: high social pressure towards smokeless tobacco use (AOR = 1.73, 95% CI: 1.21, 2.47) were significantly associated with smokeless tobacco use (Table 6).

## Discussion

The results of the study revealed that the prevalence of current users of smokeless tobacco was 45.3% among participants. The study conducted in Republic of Congo indicated, however, 18% of adults were users of smokeless tobacco [28]. This finding is higher than findings in non-pastoralist areas of Congo. The difference between the studies might be explained by the difference in culture, socio-economic and study settings (non-pastoralist). The current studies reported that large numbers of adults were user of smokeless tobacco in pastoralist communities of Ethiopia [36, 37]. This implies that lack of recognition of perceived seriousness of smokeless tobacco as a significant reason for exposing to disease, harmful to the environment, social embarrassment and family conflict, which lead to high smokeless tobacco use in this population.

This condition was reflected from IDI participants;

*“Although I don’t like chewing tobacco, I feel it’s normal. As you see it is very common in our community, it’s normal for people to chew tobacco.” [−−43-year-old health care provider]*

In this study, adults mentioned many reasons that made them to start SLT use. About 21 (20.8%) of them reported that they started SLT use from their close friends, 7.9% from family circle, 6.8% due to illness, debut 2.7% from neighbor elders, 1.4% due to sorrow and 5.7% due to others reasons.

This condition was reflected from IDI participants;

*“Chewing is our traditional habit that we follow when we grow old; it can help to avoid mouth and teeth diseases.” –56 years old male SLT user*

*“I think chewing helps us care for our teeth, I’ve tried to chew tobacco but I always got dizzy, then I stopped. I sometimes put a small amount of tobacco to crush with my teeth when I have toothache.” --41-year-old female SLT user*

*“When I was young my father asked me to buy smokeless tobacco for him. Every time I bought smokeless tobacco I lit them for him and I sometimes put the smokeless tobacco in my mouth, I did this repeatedly. I liked smokeless tobacco and I saved some money to buy smokeless tobacco for myself and hide them from people in my family and my village. I would find secret places to chew tobacco, later after getting married and having children I dared to chew tobacco in public.” --64-year-old male SLT user*

Muslims and Christians were 79 and 62% less likely to use SLT (AOR = .21, 95% CI: .13, .33) and (AOR = .38, 95% CI: .22, .67) as compared to Wakefata respectively. A possible reason for this finding may be due to local tradition and culture that influence pastoralist communities using smokeless tobacco. Moreover, Wakefata, indigenous religion in the Oromo communities, is most common among Borena pastoralist communities. Borena pastoralist community believe that smokeless tobacco which locally called ‘Derara’ is highly respected in their culture and used on different public ceremonies such as asking woman for marriage, power handover in Gada system (the Gada system is a complex institutional organization that embraced the Oromo peoples’ political, social, economic and religious life in entirety), wedding ceremony, during new baby born etc. This implies that culturally, smokeless tobacco is highly respected and has great value in this community.

This condition was reflected from IDI participants;

*“Using smokeless tobacco became a habit a long time ago. In our society, people serve chewable tobacco to guests; for instance, chewable tobaccos are served in wedding ceremonies, new baby born and traditional ceremonies.” – 30 –years-old health care provider*

*“I think chewing tobacco is a normal habit, it is commonly accepted in our traditional instructions for women to symbol of respect to welcome or greet guests who come to their house. When men visit to their house, women serve them smokeless tobacco. So to be perfect men a long time ago, men chewed.” --56-year-old man SLT user*

This finding showed that adults who believed that smokeless tobacco use is harmful to health were less likely use smokeless tobacco currently. The perception that smokeless tobacco use was harmful to health remained negatively associated with current use of smokeless tobacco use (AOR = .49, 95% CI: .34, .70). This finding is in line with study conducted in Republic of Congo [23]. This implies that adults who perceived tobacco as harmful to their health might do so to all forms of tobacco.

This condition was reflected from IDI participants;

*“I hate using smokeless tobacco because the tobacco smell makes me sick, like having nausea and headache.” --36-yearold-male non-SLT user*

*“I don’t like using smokeless tobacco because it makes chewers sick and dirty, my uncle who is a chewer coughs every day, it resembles like had tuberculosis and his body is full of tobacco smells, he always spits his saliva.” --41-year-old male non-SLT user*

This finding showed that adults who thought that smokeless tobacco use was favorable were two times more likely to be currently using smokeless tobacco. (AOR = 2.12, 95% CI: 1.48, 3.04). The possible reasons for this finding might be due to personal believe and lack of recognition of seriousness of SLT use. Moreover, the community thought that SLT is not inhaled as cigarette smoking so that STL use has no adverse effect. However, the study conducted in Bangladesh showed that compared to non-users, SLT users were less likely (*p* < 0.05) to believe that diseases were associated with SLT use [38]. The difference between the studies might be explained by community awareness and knowledge on effect of SLT use on their health. This implies that SLT use is highly influenced by personal attitude and knowledge in this population.

This condition was reflected from IDI participants;

*“I have chewed tobacco for many years, but I did not have any sickness. I saw the older generation, my grandmother, my mother, they chew tobacco, and they did not have any disease, in fact their teeth stayed very strong until they passed away.” --50-year-old male SLT user*

*“Chewing tobacco would cause no harm as it does not have smoke, it just stays in the mouth and then they spit it out.” --43-year-old female- SLT user*

This finding showed that adults who had high social pressure towards smokeless tobacco use were nearly two times more likely to use smokeless tobacco as compared to adults who had low social pressure towards smokeless tobacco use (AOR = 1.73, 95% CI: 1.21, 2.47). Possible reason for this finding might be due to perception that social norms are what an individual believes others are doing or would approve of play an important role in SLT use. This implies that social norms are important determinants of healthy behaviors in this community.

This condition was reflected from IDI participants;

*“I think that there is no benefit from chewing tobacco, but people just do it as habit, they saw others do it so they follow them, and then it becomes their habit.” --35-years-old male SLT user*

The main strength of this study is that being among pastoralist community and the first research in Ethiopia on smokeless tobacco use. Limitation of this study is that in addition to the cross sectional design, study conducted on pastoralist communities in Borena zone and cannot be generalized to all communities in Ethiopia.

## Conclusion and recommendation

Smokeless tobacco use (chewing) is very common in the selected districts of the Borena zone. The practice is strong linked to the lifestyle of the community. With regard to attitudes and social norms of community toward smokeless tobacco use, there is strong positive association on smokeless tobacco use. Being Muslim or Christian and having good perception toward the use of smokeless tobacco may be a protective factor, which prevents adults from using up smokeless tobacco. In addition, community adults are unaware of risks related to tobacco chewing.

Therefore, we recommend that smokeless tobacco control policy should be set at national level and encourages local health departments to work with tobacco control coalitions to educate the public on the negative health consequences of smokeless tobacco products. Health improvement strategies focusing on information, education, and communication on Smokeless Tobacco use is essential to help community change their practices and behaviors. In the community at large, meetings or seminars with religious leaders and ‘Aba Gadas’ (Fathers of the ‘Gada’, equivalent of president in modern governments) should be promoted as a way of short term intervention to discuss the consequences of using Smokeless tobacco. Community members should discourage harmful traditional practices those promoted Smokeless tobacco use

District health offices and other responsible bodies should expand anti-tobacco media campaigns through all possible means, such as mass media (TV, radio) and inter-personal communication (IPC) like community group discussion, men’s clubs, and women’s clubs to discuss smokeless tobacco use in the community. The capacity of existing community volunteers should be built to provide education on tobacco and help community people quit using smokeless tobacco. In addition, tobacco control in the community should start with the fundamental steps of educating the community on smokeless tobacco use-related diseases.

Community should be encouraged to work together to prevent using smokeless tobacco initiation and cessation. Capacity building should focus on skills to analyze the impact of smokeless tobacco use on the health and economy of individuals, families and the community at large. Community level tobacco control should focus on changing people’s knowledge, attitudes, perceptions and social norms with regard to smokeless tobacco use.

Health care providers should provide Health Education for adults with current use of smokeless tobacco use., Health care providers need to be aware and pro-active in prevention and cessation activities; Health messages on chewing tobacco should be tailored to the local context, and proper help should be provided to help people quit chewing; Community people should be trained on knowledge and skills to quit using smokeless tobacco. Further research focusing on health effect of chew and spit tobacco should be conducted.

## Abbreviations

CTCA: Tobacco Control in Africa
ETB: Ethiopian Birr
FMoH: Federal Minister of Health
GATS: Global Adult Tobacco Survey
GTSS: Global Tobacco Surveillance System
HHs: Households
ICD: International Classification of Diseases
LMICs: Low and middle income countries
SLT: Smokeless tobacco
ST: Smokeless tobacco
TSNAs: Tobacco-specific nitrosamines
USD: United State Dollar
WHO: World Health Organization
